# Shape and Composition Evolution in an Alloy Core–Shell Nanowire Heterostructure Induced by Adatom Diffusion

**DOI:** 10.3390/nano13111732

**Published:** 2023-05-25

**Authors:** Delong Han, Wenlei Tang, Naizhang Sun, Han Ye, Hongyu Chai, Mingchao Wang

**Affiliations:** 1 Shandong Computer Science Center (National Supercomputer Center in Jinan), Qilu University of Technology (Shandong Academy of Sciences), Jinan 250014, China; handl@qlu.edu.cn; 2 State Key Laboratory of Information Photonics and Optical Communications, Beijing University of Posts and Telecommunications, Beijing 100876, China; tang2021110591@bupt.edu.cn (W.T.); snz@bupt.edu.cn (N.S.); han_ye@bupt.edu.cn (H.Y.); 3 Key Laboratory of Semiconductor Materials Science, Beijing Key Laboratory of Low Dimensional Semiconductor Materials and Devices, Institute of Semiconductors, Chinese Academy of Sciences, Beijing 100083, China; 4 Centre for Theoretical and Computational Molecular Science, Australian Institute for Bioengineering and Nanotechnology, The University of Queensland, St Lucia, QLD 4072, Australia

**Keywords:** nanowire, core–shell heterostructure, growth model, morphology, alloy composition

## Abstract

A core–shell nanowire heterostructure is an important building block for nanowire-based optoelectronic devices. In this paper, the shape and composition evolution induced by adatom diffusion is investigated by constructing a growth model for alloy core–shell nanowire heterostructures, taking diffusion, adsorption, desorption and incorporation of adatoms into consideration. With moving boundaries accounting for sidewall growth, the transient diffusion equations are numerically solved by the finite element method. The adatom diffusions introduce the position-dependent and time-dependent adatom concentrations of components A and B. The newly grown alloy nanowire shell depends on the incorporation rates, resulting in both shape and composition evolution during growth. The results show that the morphology of nanowire shell strongly depends on the flux impingement angle. With the increase in this impingement angle, the position of the largest shell thickness on sidewall moves down to the bottom of nanowire and meanwhile, the contact angle between shell and substrate increases to an obtuse angle. Coupled with the shell shapes, the composition profiles are shown as non-uniform along both the nanowire and the shell growth directions, which can be attributed to the adatom diffusion of components A and B. The impacts of parameters on the shape and composition evolution are systematically investigated, including diffusion length, adatom lifetime and corresponding ratios between components. This kinetic model is expected to interpret the contribution of adatom diffusion in growing alloy group-IV and group III-V core–shell nanowire heterostructures.

## 1. Introduction

Semiconductor nanowires have attracted extensive attention due to their excellent photoelectric properties and great application potential [1,2,3,4]. When heterostructures are formed by nanowires, the interactions of the low-dimensional components and their interfaces can give rise to optical emission, carrier transport, and other properties [5]. An axial nanowire heterostructure (NWH) is formed when the composition is modulated along the long axis of the nanowire, while the composition of the radial (core–shell) NWH is modulated orthogonal to the long axis. Compared with axial NWHs, core–shell NWHs can be made in a wider range of material systems, because radial growth is essentially thin-film deposition on a NW substrate. Nowadays, core–shell NWHs have been applied in photonic crystals, solar cells, light-emitting diodes, photodetectors, and thermoelectric devices [6,7,8]. To achieve precise control of the morphology and composition distribution of core–shell NWH, research on the growth mechanism is necessary [9].

MBE (molecular beam epitaxy), CVD (chemical vapor deposition), and MOCVD (metal organic chemical vapor deposition) are the main approaches for the preparation of core–shell NWHs. According to the different growth methods, there are three main growth mechanisms including VLS (vapor–liquid–solid), VSS (vapor–solid–solid), and VS (vapor–solid). Different vapor phase materials can be deposited on the sidewall of NWs to realize radial heteroepitaxial growth [10]. L. J. Lauhon et al. [11] grew Si-Ge core–multishell NWHs by repeated modulation of reactants in CVD, which had been used to build the coaxially gated NW field-effect transistor. N. Sköld et al. [12] synthesized GaAs−GaInP core−shell NWHs by metal−organic vapor phase epitaxy (MOCVE). The GaInP shell had a tapered shape and a Ge concentration between 0.34 to 0.69. GaAs-GaAsP core–shell NWHs can also be synthesized by MOCVE [13]. S. Assali et al. [14] synthesized Ge-GeSn core–shell NWH arrays by CVD. The GeSn shell had Sn incorporation of up to 13% without the formation of Sn clusters and structural defects, which resulted in a direct band gap room temperature emission at 0.465 eV. D. C. Dillen et al. [15] synthesized Ge-SiGe NWHs with a modulation-doped shell by CVD. Modulation doping element B was combined as an acceptor with epitaxial SiGe shell growth. O. Hayden et al. [16] grew a CdS shell on a Si NW using pulsed-laser deposition (PLD). The Si-CdS NWH was used to fabricate lithographically defined intrawire p–n junctions via a selective etching process. Moreover, the preparation of Sb2Te3/GeTe, InP/Ga2O3, GaP/GaN, and other core–shell NWHs has also been realized [17,18,19].

The morphology and composition distribution of NWHs has a significant impact on the optical, electrical, mechanical, and other properties [20]. Researchers have observed tapered nanowires and NWHs in growth experiments [21,22,23]. J.W. Lee et al. [21] found that Si-Ge core–shell NWHs grown using Au-catalyzed CVD had tapering shape edges. The TEM images showed that the diameters of the top, middle, and bottom of the NWHs were 20 nm, 80 nm, and 800 nm, respectively. H. L. Zhou et al. [22] and J. H. Kang et al. [23] observed tapered GaAs-AlGaAs core–shell NWHs which were grown by different methods (Au-assisted MBE and Au-assisted MOCVD). Tapered NWHs can be caused by the morphological changing of catalyst droplets or the inhomogeneous radial deposition of vapor material. There has been some theoretical research on the growth mechanism of nanowires. Koryakin et al. [24] studied the growth of InAs nanowires based on the classical nucleation theory and found that elastic stress can affect the growth rate of nanowires. Ab initio simulations have also been successfully used for simulations of the growth and properties of NWHs [25,26]. M. Alves Machado Filho et al. [26] studied the formation mechanism of InAlN core–shell NWHs by addressing precursor prevalence and energetics using the DFT-based synthetic growth concept. They found that the formation of the core–shell structure is substantially driven by the precursors’ abundance and their preferential bonding onto the growing edge of the nanoclusters/islands. The composition distribution of alloyed NWHs is complicated in the experiment. S. Assali et al. [14] observed enhanced Sn incorporation stripes on the {112} side-facets in the GeSn shell of Ge-GeSn core–shell NWHs. Q. Li et al. [27] analyzed the composition distribution of GaN-InGaN core–shell NWHs through EDXS and found that the composition increases from 20% near the InGaN/GaN interface to 40% near the InGaN surface. There are also some theoretical studies on the composition distribution of NWHs. Ye et al. [28] established a model to explain the axial composition evolution in multi-component alloy nanowires based on the kinetic framework and the processes of diffusion and adsorption. Yao et al. [29] studied the phase separation mechanism on the surface of catalyst particles based on the first principles and put forward the prerequisite for the catalytic growth of nanowires. Zhang et al. [30] studied the spontaneous formation mechanism of Al-rich radial fringes in GaAs-AlGaAs core–shell NWHs based on the diffusion process, attributing the inhomogeneous composition distribution to the different atom mobility in different crystal directions. V. G. Dubrovskii et al. [31] studied the influence of lateral growth on nanowires’ shape by solving the steady-state diffusion equation on the nanowire sidewall. The adatom concentration distribution on NW lateral faces is taken into consideration. The results of the NW growth theoretical model were in good agreement with the growth experiment on InAs NWs. The concentrations of adatom at the top and the bottom of the NWH are assumed constant, and the diffusion equation was only solved on the NWH sidewall. G. Vastola et al. [32] built a theoretical model which treats the atomic exchange between the surface and the sub-surface and the driving force for surface segregation as the three key parameters that control the interface composition profile of core–shell NWHs. This model explained the abrupt and diffuse interface composition profiles of core–shell NWs. However, adatom surface diffusion is not considered which results in an identical shape and composition along the nanowire. These existing theoretical works studied the morphology and composition of core–shell NWHs separately and did not include the contribution of adatom diffusion. Although some phenomena in the growth of NWH had been explained, the influence of adatom diffusion on the morphology and composition of core–shell NWHs is still unclear. Hence, it is necessary to study the evolution of composition distribution in core–shell nanowire heterostructures coupled with the change in morphology.

In this paper, the morphology and composition evolution of core–shell NWHs is numerically simulated based on adatom diffusion equations. Different diffusion parameters are taken into consideration. The evolution of composition distribution in core–shell NWHs is studied coupled with the change in morphology. All the analyses in this paper are based on the finite element method.

## 2. Modelling

**Figure 1:**
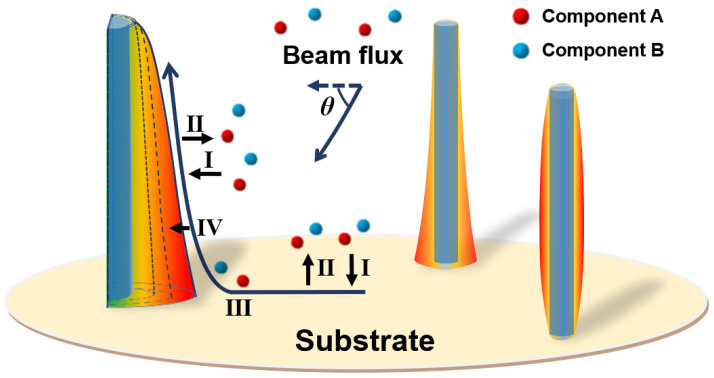
Growth model of alloy core–shell nanowire heterostructure, including the adsorption, diffusion, and incorporation of components A and B.

To unveil the diffusion-induced shape and composition evolution of core–shell NWHs, a general growth model was constructed based on adatom diffusion as illustrated in Figure 1. The alloy shell will grow on the nanowire sidewall under the beam flux of two components denoted by A and B. Four major processes are taken into consideration, including (I) adsorption on the NWH sidewall and the substrate, (II) desorption on the NWH sidewall and the substrate, (III) diffusion of adatoms on the NWH sidewall and the substrate, and (IV) incorporation of adatoms on the NWH sidewall to form the growing epitaxial layer. Due to the non-uniform growth velocity of the sidewall, the morphology of the core–shell NWH may be prominent or cupped as shown in Figure 1. Meanwhile, the composition distribution in the shell varies along both the axial and radial directions of the nanowire. The adatom concentration of component A and component B on the substrate and the NWH sidewall can be obtained by solving the following time-dependent diffusion equations:

(1) $$\frac{\partial c_{i,s}}{\partial t}={𝛻}_{t}\cdot \left(D_{i,s}{𝛻}_{t}c_{i,s}\right)+F_{i,s}-\frac{c_{i,s}}{\tau_{i}^{des}}$$

(2) $$\frac{\partial c_{i,sw}}{\partial t}={𝛻}_{t}\cdot \left(D_{i,sw}{𝛻}_{t}c_{i,sw}\right)+F_{i,sw}-\frac{c_{i,sw}}{\tau_{i}^{in}}-\frac{c_{i,sw}}{\tau_{i}^{des}}$$

where $c_{i,x}$ is the adatom concentration. The subscripts x=s and x=sw denote the substrate and the sidewall, respectively. *i* denotes component A or B. $D_{i,x}$ is the diffusion coefficient, defined as $D_{i,x}=\frac{\lambda_{i}^{2}}{\tau_{i}}$, where $\lambda_{i}$ is the diffusion length of the adatom. We suppose that the adatom lifetime $\tau_{i}$ on the NWH surface is composed of two processes: incorporated into the solid shell as the outermost layer and desorbed back from the surface to vapor. The two processes can be expressed as $\frac{1}{\tau_{i}}=\frac{1}{\tau_{i}^{in}}+\frac{1}{\tau_{i}^{des}}$, where $\tau_{i}^{in}$ and $\tau_{i}^{des}$ denote the incorporation and desorption, respectively. $\tau_{i}^{in}$ and $\tau_{i}^{des}$ are defined as $\tau_{i}^{in}=\left(\zeta +1\right)\tau_{i}$, $\tau_{i}^{des}=\frac{\tau_{i}^{in}}{\zeta }$. $F_{i,x}$ is the adatom supplement rate from vapor. To vary the ratio between the adsorption flux on the sidewall and the substrate, we define *θ* as the angle between the beam flux and the direction perpendicular to the nanowire. In this sense, $F_{i,sw}=F_{i}\cos(\theta )$ and $F_{i,s}=F_{i}\sin(\theta )$. For simplicity, only the initial cylindrical nanowire core without the taper is modeled. Complicated morphology such as hexagonal nanowires needing 3D models are not included. Moreover, we ignored the shadow effect in the MBE, and only the core–shell nanowire with a symmetric shell is considered.

To solve the diffusion equations, we adopt the following boundary conditions:

(3) $${\left.\frac{\partial c_{i,s}}{\partial r}\right|}_{r=R_{s}}=0$$

(4) $$D_{i,s}{\left.\frac{\partial c_{i,s}}{\partial r}\right|}_{r=R_{nw}}=-D_{i,nw}{\left.\frac{\partial c_{i,nw}}{\partial z}\right|}_{z=0}$$

(5) $${\left.c_{i,nw}\right|}_{z=L}=0$$

Equation (3) is a far-field condition indicating the concentration far from the NWH, i.e., at the edge of our simulation cell (*r* = *R_S_*). Equation (4) describes the continuity of mass at the bottom of the NWH where the substrate meets the sidewall. Equation (5) implies that all adatoms diffused to the top of the NWH will be absorbed by the catalyst. Since we focus on the radial growth of the NWH, the axial growth due to the catalyst is ignored. Meanwhile, we set the concentration on the top of the nanowire to zero rather than an assumed constant [32]. Based on the time-dependent diffusion equations, we can obtain the growth velocity of the nanowire lateral surface for each component by mass conservation:

(6) $$v_{i}\left(z,t\right)=\frac{c_{i,s}\left(z,t\right)\cdot {\left(\frac{a_{i}}{2}\right)}^{2}}{2\pi R\left(z,t\right)\cdot \tau_{i}^{in}}$$

where $a_{i}$ is the lattice constant of component *i*, and $R\left(z,t\right)$ is the instantaneous radius of the NWH shell which should follow:

(7) $$\frac{\partial R\left(z,t\right)}{\partial t}=v_{radial}=\sum v_{i}\left(z,t\right)$$

The above time-dependent differential equations were numerically solved by the finite element method implemented in Comsol Multiphysics. An arbitrary Lagrangian–Eulerian (ALE) method was employed to move the NWH sidewall at the velocity given by $v_{radial}$.

After obtaining each growth rate of components A and B, we can proceed on to investigate the composition profile in the alloyed NWH shell. The surface composition (ξ, fraction of *A*) at the NWH sidewall during growth can be described by the following equation:

(8) $$\delta \frac{\partial \xi \left(z,t\right)}{\partial t}=v_{A}\left(z,t\right)-\xi \left(z,t\right)\Sigma v_{i}\left(z,t\right)$$

where δ is the thickness of the surface layer into which the adatoms incorporate. We assume that δ does not change with time. The bulk diffusion and exchange of incorporated atoms inside the heterostructure [33] is not taken into consideration. The strain effect induced by lattice mismatch results in the redistribution of atoms in thermodynamics [34,35,36], and it is not included as well since the kinetic of the adatoms is focused. The diffusion length and adatom lifetime are used to account for the different components, *A* and *B*, by the relation $\tau_{B}=\eta \tau_{A}$ and $\lambda_{B}=\gamma \lambda_{A}$. In this paper, we assume the default parameters as follows: $R_{ini}=50\;\mathrm{nm}$, $a_{A}=0.5\;\mathrm{nm}$, $\tau_{A}=100\;s$, ξ=1, and η=1. Seung-Hyun Lim et al. [37] found that the surface diffusion lengths of Si adatoms along the [110] direction were estimated to be about 70 nm, 140 nm, and 200 nm at 550 °C, 600 °C, and 650 °C, respectively. Considering the harmony of our model, we comparably choose $\lambda_{A}=500\;\mathrm{nm}$. Jiang et al. [38] determined the surface diffusion length of Ge adatoms on Si(001) to be 2.1 μm. γ=2 was chosen to make $\lambda_{B}$ reach the same order of magnitude. Lifetime is obtained by inverse calculation of the diffusion length and the diffusion coefficient as it cannot be obtained directly. V. G. Dubrovskii et al. [31] adopted $\frac{\tau_{i}}{\tau_{i}^{in}}=0.9$ in their growth model to reproduce the thickness of an InAs nanowire grown by MBE. Without clear experimental evidence, we set a simple ratio as 0.5 in our model.

## 3. Results and Discussion

**Figure 2:**
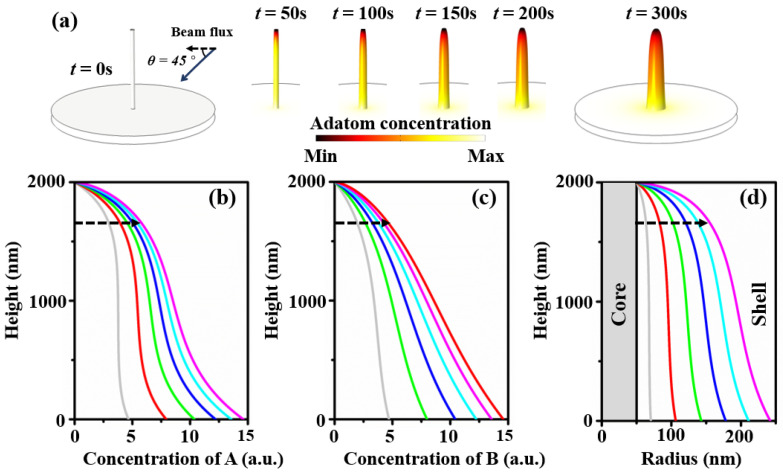
(**a**) Snapshots of the surface adatom concentration and shape evolution of core–shell nanowire heterostructures during the growth process with an impingement beam angle of 45°. (**b**) Surface concentration of component A along the nanowire sidewall. (**c**) Surface concentration of component B along the nanowire sidewall. (**d**) Radius along the nanowire sidewall.

Based on the growth model with different parameters, we found that core–shell NWHs have a certain pattern of adatom concentration distribution and shape. The diffusion length of adatom A is 500 nm, and the diffusion length of adatom B is set twice that of adatom A. Here, we take *θ* = 45° (*θ* is the beam flux impingement angle) as an example. This ensures identical deposition rates on the sidewall and substrate, accounting for MBE and MOCVD [30,35]. Thus, this can be regarded as a general case. In the MBE growth of core–shell nanowire, the nanowire stands upright on the substrate. When the beam flux hits the nanowire at a certain angle, it will naturally form a shadow area to block the adatoms hitting the substrate. In this shadow area, if the substrate cannot receive adatoms, it will affect the adatoms diffusing from the substrate to the nanowire. This will lead to asymmetry of the nanowire’s growth [39,40]. Figure 2a shows changes in the shape of the nanowire over time, using shades of color to indicate concentration levels. The concentration distributions of adatoms A and B are shown separately in Figure 2b,c. Qualitatively, it is easy to see that the concentrations of both adatoms follow the same trend, with them being smaller at the top of the nanowire and increasing at the lower section. The quantification has a difference in shape due to the different diffusion lengths. Figure 2d shows the evolution of the nanowire shell over time. The middle of the nanowire reached a radius of 192 nm after 300 s of growth.

### 3.1. Shape Evolution

**Figure 3:**
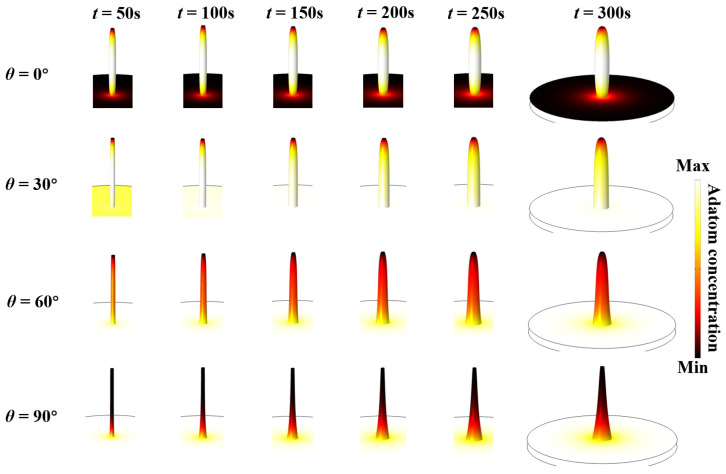
Snapshots of the surface adatom concentration and shape evolution of the core–shell nanowire heterostructures during the growth process with various impingement beam angles.

We previously focused on the impingement beam angle *θ* equal to 45° as an exemplary case. In order to further investigate the effect of *θ* on the evolution of the shape, more values of *θ* will be investigated, as well as a more detailed delineation of the nanowire shape. The diffusion length of adatom A (500 nm) is half that of adatom B. *θ* controls the ratio of sidewall to substrate adsorption, and adjusting different *θ* results in different nanowire morphologies. Adatoms with shorter diffusion lengths tend not to diffuse far away from the position where the beam flux hits the nanowire. Here, we have exhibited morphologies at different time points for *θ* values of 0°, 30°, 60°, and 90° as shown in Figure 3. For *θ* = 0°, the nanowire is thick in the middle and thin at the two ends. As for the other values of *θ*, the nanowires are all thinner at the top and thicker at the bottom. The radius increases faster at the bottom with a lager *θ*. We can see that for *θ* = 0°, the nanowire is white in the middle and black at the bottom and top, which means component A diffuses from the middle to the ends. For the other angles, component A diffuses from the bottom to the top. This is also the reason for the changes in the distribution of the white parts as shown in Figure 3.

**Figure 4:**
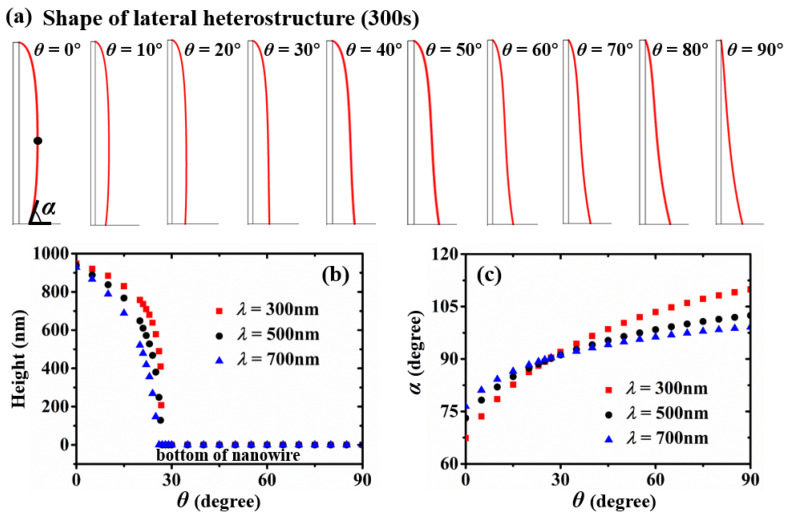
(**a**) Shape of the core–shell nanowire heterostructure after 300 s growth with impingement beam angles from 0° to 90°. (**b**) Relationship between the position of the maximum radius on the nanowire and the impingement beam angle. (**c**) Relationship between the bottom contact angle and the impingement beam angle.

To investigate the relationship between the beam flux impingement angle and the shape evolution more quantitatively, we systematically mapped growth models with the value of *θ* changes from 0° to 90° at 10° intervals, as shown in Figure 4a. In the range $10–25\;eV^{-1}$ for $1/k_{B}T$, between 192 °C and 888 °C, C.M. Aldao et al. [41] found that, for different diffusion models, diffusion coefficients of a Si adatom on Si(100) are different. For open circles, solid circles, and open triangles, $D=1.29\times 10^{-7}exp\left(\frac{-0.239eV}{k_{B}T}\right)$, $0.8\times 10^{-7}exp\left(\frac{-0.236eV}{k_{B}T}\right)$, and $0.07\times 10^{-7}exp\left(\frac{-0.598eV}{k_{B}T}\right)$, respectively. Comparably, we simulated the influence of different diffusion lengths on the shape evolution. Here, we selected 300 nm, 500 nm, and 700 nm, defining the position of the maximum nanowire radius. As shown in Figure 4b, the thickest position of the nanowires with different diffusion lengths all drop to the bottom before 30°. This position of the nanowires grown by the adatoms with longer diffusion lengths will drop at a smaller impingement angle. We define the bottom contact angle between nanowire with substrate as α. There is a critical value of the impingement beam angle. When *θ* is below 26°, α is an acute angle. With a longer diffusion length, a larger α will be formed. When *θ* is above 26°, α is an obtuse angle, and it has the contrary relationship to the diffusion length as before.

Parameters other than the impingement beam angle *θ* mainly affect the size of the radius and the exact quantitative relationship can be seen in the Supplementary Materials. In Figure S1, when *θ* is set to 0° or 90°, a longer diffusion length results in thicker growth of the nanowire. *θ* = 45° produces a cross-over in the middle section of the nanowire. The upper section of the nanowire grows thicker with a smaller diffusion length, while the situation is the opposite for the lower section of the nanowire. As shown in Figure S2, lifetime τ is inversely proportional to the radius. The situation is similar to the fraction of component *A*, whereby the nanowire tends to grow thinner with greater ξ, as shown in Figure S3. As for the ratio of $\lambda_{B}$ to $\lambda_{A}$, the radius increases with the increased γ, as shown in Figure S4.

### 3.2. Composition Evolution

**Figure 5:**
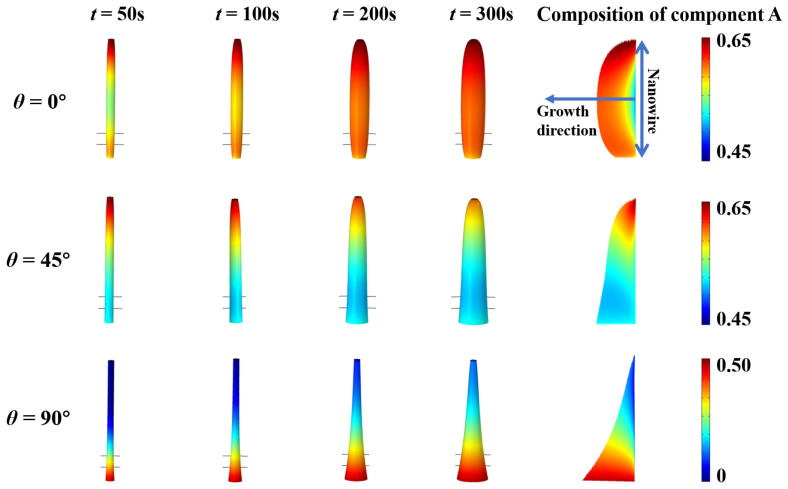
Snapshots of the surface composition of component A along with the shape evolution of the core–shell nanowire heterostructures during the growth process with various impingement beam angles.

Along with shape evolution, the diffusion of adatoms will introduce a non-uniform composition profile for components *A* and *B*. Here, we investigate the surface composition of core–shell nanowire heterostructures in the growth process and demonstrate the accumulated composition in both radial and axial directions. The simulations are performed with the default parameters, i.e., $\lambda_{A}=500\;\mathrm{nm}$, $\lambda_{B}=1000\;\mathrm{nm}$, $\tau_{A}=100\;s$, and $\tau_{B}=100\;s$. Three typical beam flux impingement angles 0°, 45°, and 90° are considered as exemplary cases. The surface composition profile of component *A* are shown in Figure 5. For *θ* = 0°, the composition remains at the maximum at the top and has a non-monotonic variation downwards. The composition of *A* in the nanowire is mostly bigger than 0.5. We attribute this to the longer diffusion length of component *B*, which leads to the higher percentage of component *B* diffusing off the nanowire. For *θ* = 45°, part of adatoms in the beam flux adsorbs onto the substrate, and these adatoms will diffuse to the nanowire. This results in a decrease in the composition of *A* in the middle range of the nanowire, and a little rebound at the bottom. When we recall the concentration distribution of adatoms *A* and *B* shown in Figure 2, the change in the slope of the curve for component *B* is much lower than that of component *A*. Theoretically, it is consistent with the current composition of component *A*. For *θ* = 90°, the composition shows a monotonic increase from the bottom to the top, since all of the adatoms diffuse upward from the substrate to the top of nanowire. Component *B* has an advantage to diffuse to the upper part; thus, the overall value of the composition of component *A* does not exceed 0.5. Snapshots of the surface composition profile for the other beam flux impingement angles are demonstrated in Figure S5. The compositions of component *A* accumulated over 300 s are shown on the right side in Figure 5. Since we focused on the composition induced by adatom diffusion, the bulk diffusion and exchange in the solid nanowire are ignored. For *θ* = 0°, the composition of *A* increases gradually from the core to the shell in the radial direction. For *θ* = 45°, the composition of *A* decreases from the top to the bottom, which is the opposite to that of *θ* = 90°.

**Figure 6:**
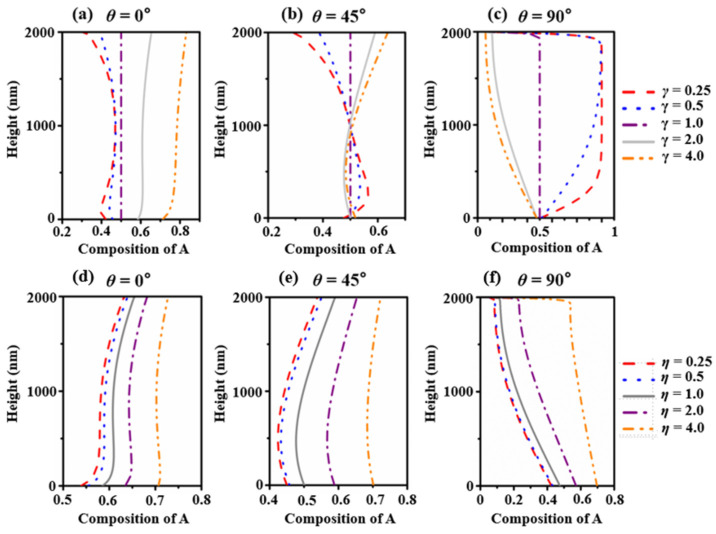
(**a**) *θ* = 0°, (**b**) *θ* = 45°, and (**c**) *θ* = 90°. Impact of the lifetime ratio between components A and B on the surface composition after 300 s of growth with the impingement beam angles (**d**) *θ* = 0°, (**e**) *θ* = 45°, and (**f**) *θ* = 90°.

V. Consonni et al. [42] simulated the growth of GaN NWs grown on a Si(111) substrate by using plasma-assisted MBE and set the efficient diffusion length as 45 nm. Z. Y. Zhong et al. [43] estimated the surface diffusion coefficient of Ge on a Si(001) substrate at temperatures of 600 °C, 690 °C, 720 °C, and 800 °C to be 350, 440, 440, and 440, respectively. Obviously, different adatoms have very different diffusion coefficients. Previously, we confirmed that the diffusion coefficient has an effect on the shape and composition evolution. In this work, we hope to further study the effects of the two parameters that affect the diffusion factor. We distinguish the differences of components *A* and *B* by the parameters *γ* and *η* accounting for the different diffusion lengths and adatom lifetimes; we define $\gamma =\frac{\lambda_{B}}{\lambda_{A}}$, $\eta =\frac{\tau_{B}}{\tau_{A}}$. We maximize the difference by adjusting the ratio. The surface composition profiles of different γ and η are shown in Figure 6. The composition calculated by the default values are drawn with solid lines. When γ=1, the composition should maintain a constant value at 0.5 as a validation because we are treating a non-alloy case. For *θ* = 0°, when γ<1, the change trend in the composition appears non-monotonic. The composition of component A increases at first and then decreases with one rebound as the axial height decreases. When γ>1, the composition lines increase monotonically from the bottom to the top. For *θ* = 45°, when γ<1, the composition increases from the top to the bottom with a decrease immediately, which is the opposite to that of larger γ. Qualitatively, the component with the smaller diffusion length segregates on the top. For *θ* = 90°, when γ>1, the composition lines monotonically increase from the bottom to the top. When γ<1, the composition lines increase from the bottom and end with a rapid reduction in the top. The component with the larger diffusion length segregates on the top. All of the composition lines of component A have the same trend in that the overall value increases with η. For each set of curves, we observe from the bottom up. For *θ* = 0°, the curves are monotonically increasing, with a rapid increase in the middle and a decrease in the rate of the increase at the two ends. For *θ* = 45°, the curves decrease a little and then maintain their increase. For *θ* = 90°, the curves have a monotonic decrease; a smaller η has a faster decrease rate.

## 4. Conclusions

In summary, we construct a transient growth model for alloy core–shell nanowire heterostructures to investigate the shape and composition evolution induced by adatom diffusion. The growth model includes the adatom diffusion, adsorption, desorption, and incorporation in the nanowire. The shape and composition evolution are demonstrated by solving the diffusion equations using the finite element method with moving boundaries. The radius of the nanowire heterostructure is non-uniform along the nanowire and the morphology strongly depends on the flux impingement angle. The position of the largest radius moves from near the middle of nanowire to the bottom, with an increasing impingement angle. Since diffusion determines the surface adatom concentration, the competition between components A and B results in a non-uniform composition profile along with the shape evolution. The impacts of the parameters on the shape and composition evolution, including diffusion length, adatom lifetime and corresponding ratios, are demonstrated. We expect these results can help to develop a fundamental understanding of group-IV and group III-V core–shell nanowire heterostructures.

## Data Availability

The data presented in this study are available on request from the corresponding author.

## Supplementary Materials

The following supporting information can be downloaded at: https://www.mdpi.com/article/10.3390/nano13111732/s1.
