# Supplementary material for: Shape and Composition Evolution in an Alloy Core–Shell Nanowire Heterostructure Induced by Adatom Diffusion
Source: Nanomaterials (Basel). 2023 May 25;13(11):1732. doi: 10.3390/nano13111732 (PMC10254845; doi:10.3390/nano13111732)
Supplement: Supplementary file 1 [file nanomaterials-13-01732-s001.zip › nanomaterials-2381154-supplementary.pdf]

nanomaterials

Article

Shape and composition evolution in alloy core-shell nanowire heterostructure induced by adatom diffusion

Delong Han [1]#, Wenlei Tang [2]#, Naizhang Sun [2], Han Ye [2], Hongyu Chai [3]* and Mingchao Wang [4]*

1 Shandong Computer Science Center (National Supercomputer Center in Jinan), Qilu University of Technology (Shandong Academy of Sciences), Jinan 250014, China; handl@qlu.edu.cn (D.H.)
2 State Key Laboratory of Information Photonics and Optical Communications, Beijing University of Posts and Telecommunications, Beijing 100876, China; tang2021110591@bupt.edu.cn (W.T.); snz@bupt.edu.cn(N.S.); Han_ye@bupt.edu.cn(H.Y.)
3 Key Laboratory of Semiconductor Materials Science, Beijing Key Laboratory of Low Dimensional Semiconductor Materials and Devices, Institute of Semiconductors, Chinese Academy of Sciences, Beijing 100083, China
4 Centre for Theoretical and Computational Molecular Science, Australian Institute for Bioengineering and Nanotechnology, The University of Queensland, St Lucia, QLD 4072, Australia
#These authors contribute equally
* Correspondence: mingchao.wang@uq.edu.au (Dr. M. Wang); chaihongyu@semi.ac.cn (Dr. H. Chai).

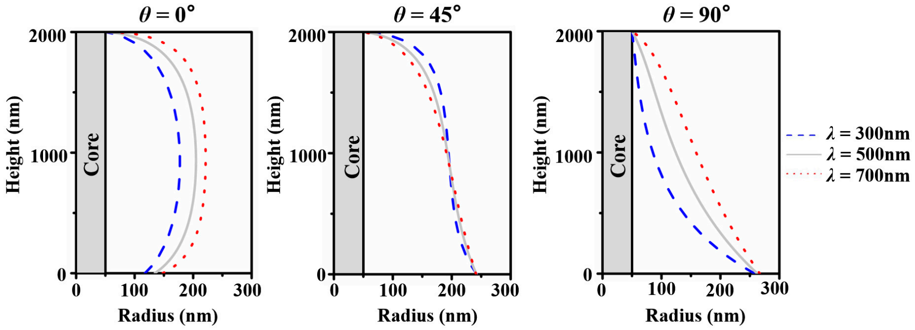

Figure S1. Relation between the shape of core-shell nanowire heterostructure and diffusion length of adatoms (component A). The growth time is set 300s.

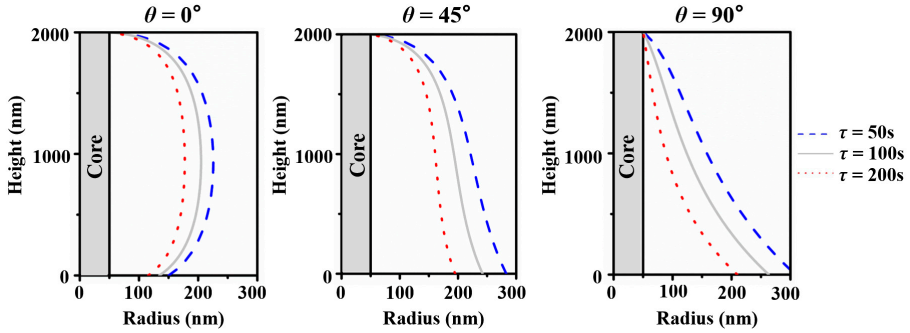

Figure S2. Relation between the shape of core-shell nanowire heterostructure and the lifetime of adatoms (component A). The growth time is set 300s.

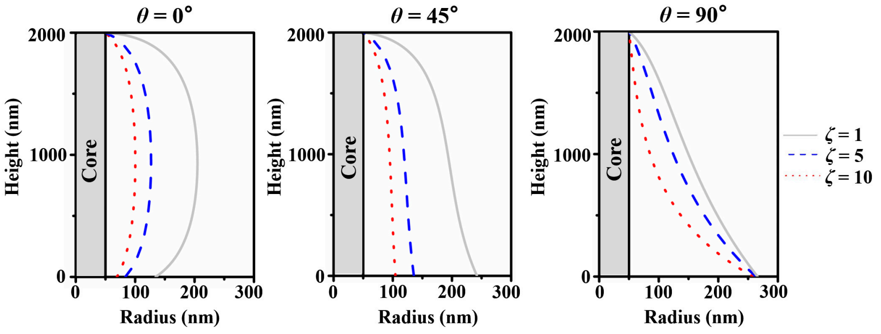

Figure S3. Relation between the shape of core-shell nanowire heterostructure and the parameter ζ. The growth time is set 300s.

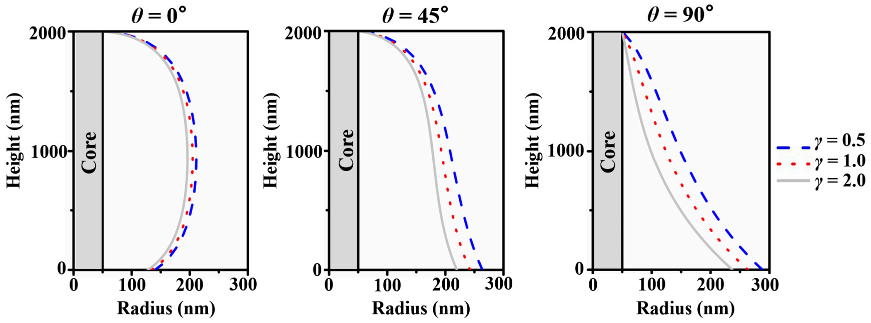

Figure S4. Relation between the shape of core-shell nanowire heterostructure and the parameter γ. The growth time is set 300s.

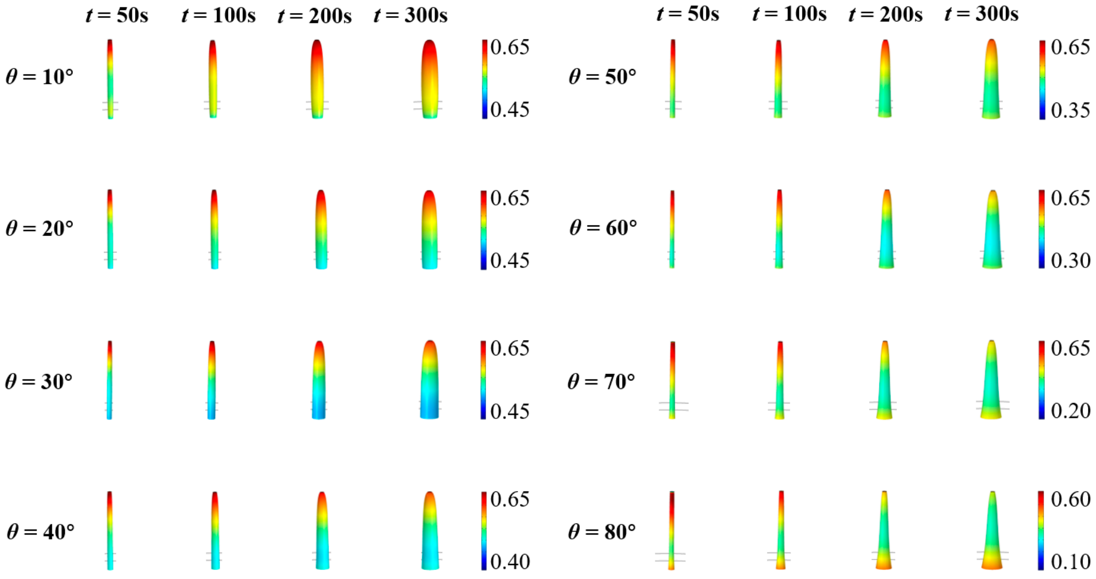

Figure S5. Snapshots of surface composition of core-shell nanowire heterostructures during growth process with different impingement beam angles.
